# Risk communication and community engagement strategies for COVID-19 in 13 African countries

**DOI:** 10.34172/hpp.2021.18

**Published:** 2021-05-19

**Authors:** Yusuff Adebayo Adebisi, Adrian Rabe, Don Eliseo Lucero-Prisno III

**Affiliations:** ^1^Global Health Focus, London, United Kingdom; ^2^African Young Leaders for Global Health, Abuja, Nigeria; ^3^Faculty of Pharmacy, University of Ibadan, Ibadan, Nigeria; ^4^Faculty of Medicine, School of Public Health, Imperial College London, London, UK; ^5^Department of Global Health and Development, London School of Hygiene and Tropical Medicine, London, UK

**Keywords:** COVID-19, Risk communication, Community engagement, Responses, Africa

## Abstract

**Background:** Coronavirus disease 2019 (COVID-19) outbreak is a major threat facing health systems globally and African countries are not an exception. Stakeholders, governments, and national authorities have mounted responses to contain the pandemic. This study aimed to catalogue the risk communication and community engagement (RCCE) strategies as well as the challenges facing RCCE in 13 African countries.

**Methods:** We conducted a narrative review of evidence to answer the aim of the study. The search was conducted in March 2021 and evidence published between December 2019 and February 2021 were included. Data reported in this article were obtained from reports, literature in peer-reviewed journals, grey literature and other data sources in 13 African countries. The 13 countries include Ethiopia, Ghana, Kenya, Algeria, Angola, Cote d’Ivoire, the Democratic Republic of the Congo, Mauritius, Nigeria, South Africa, Tanzania, Uganda, and Zambia. The authors also snowball further data to gather information for this review.

**Results:** Most of the priority African countries have RCCE strategies to contain the transmission and spread of the coronavirus. Our findings revealed RCCE strategies in the 13 African countries focused on training and capacity building, risk communication systems, internal and partners’ coordination, community engagement, public communication, contending uncertainty, addressing misperceptions and managing misinformation. However, the RCCE response activities were not without challenges, which included distrust in government, cultural, social, and religious resistance, and inertia among others.

**Conclusion:** With the similar RCCE approaches and interventions seen across the countries, it is clear that countries are learning from each other and from global health organizations to develop COVID-19 RCCE programs. It is important for African countries to address the challenges facing RCCE in order to effectively contain the pandemic and to prepare for future public health emergencies.

## Introduction

Coronavirus disease 2019 (COVID-19) outbreak is a major health threat of the21st century and Africa is not exempted.^1^ Countries around the world have mounted responses to contain the pandemic.^2^ As of 14 February 2020, it was reported that the outbreak had spread to Egypt in Africa.^3^ At the end of February, Nigeria reported the first COVID-19 case in sub-Saharan Africa.^3^ Most of the imported cases on the African continent arrived from countries outside the region (Europe and United States) and not China where the virus originated.^4^ As of 18 March 2021, 4 106 497 cases and 109 146 deaths have been recorded in Africa.^5^ It is believed that many COVID-19 cases go unreported in many African countries which is attributed to their peculiarly weak healthcare systems, limited infrastructure and equipment, limited funding, inadequate training of health workers, and substandard transmission of data for effective surveillance among other fundamental challenges.^1^ Nonetheless, there has been a rapid response to contain the pandemic by many African countries long before cases were reported.^6^

The Africa Centres for Disease Control and Prevention (Africa CDC) started the Africa Task Force for Novel Coronavirus (AFCOR) on 3 February 2020.^1^ AFCOR has been engaging with World Health Organization (WHO) African Region on risk communication and community engagement (RCCE), surveillance, including contact tracing and points of entry screening, diseases prevention and control in healthcare facilities, laboratory diagnosis and clinical management of COVID-19 patients.^7^ Until vaccines or pharmacological treatments are developed and widely implemented, it is therefore pertinent that behavioural changes and willingness to obey precautionary measures remain the main powerful tools to respond to the pandemic.^8^ This makes it important to examine and emphasize the role RCCE plays in stopping the chains of transmission and mitigating the impact of the outbreak.^8^

RCCE refers to “the processes and approaches to systematically consult, engage, and communicate with communities who are at risk, or whose practices affect risk”.^9^ The aim of RCCE is to urge, enable and include stakeholders in the prevention of and response to public health emergencies by adapting communication to community actualities.^10^ For COVID-19, RCCE enables stakeholders to work hand-in-hand to ensure healthy behaviour and reduce the risk of transmitting and spreading the coronavirus.^10^ Integrating RCCE into the national public health emergency response is imperative.^8^ In 2020, the WHO provided guidance on RCCE for countries to help protect the people’s health in response to the outbreak.^10^ Actionable plans were recommended on how to develop effective RCCE strategies in preparation for the outbreak. However, unproductive RCCE in some African countries still threatened effective response to the pandemic.^11^

The COVID-19 outbreak emphasized that the most important and effective interventions in public health response to outbreaks is proactive and effective communication. With the emergence of the COVID-19 pandemic, a novel infectious disease, providing accurate and timely information and countering disinformation and misinformation have never been more necessary.^12^ WHO Regional Office for Africa has partnered with the Africa CDC, United Nations Children’s Fund (UNICEF), the International Federation of Red Cross and other organizations to coordinate risk communications and community engagement in countries.^13^ A the early days of the pandemic, based on the huge number of travels and direct link to China, the WHO identified 13 priority African countries for COVID-19 which including Algeria, Ghana, South Africa, Tanzania, Kenya, Mauritius, Angola, Cote d’Ivoire, Ethiopia, the Democratic Republic of the Congo, Nigeria, Zambia, and Uganda.^7^

This study aimed to catalogue the RCCE strategies as well as the challenges facing RCCE in the 13 African countries.

## Material and Methods

**Figure 1:**
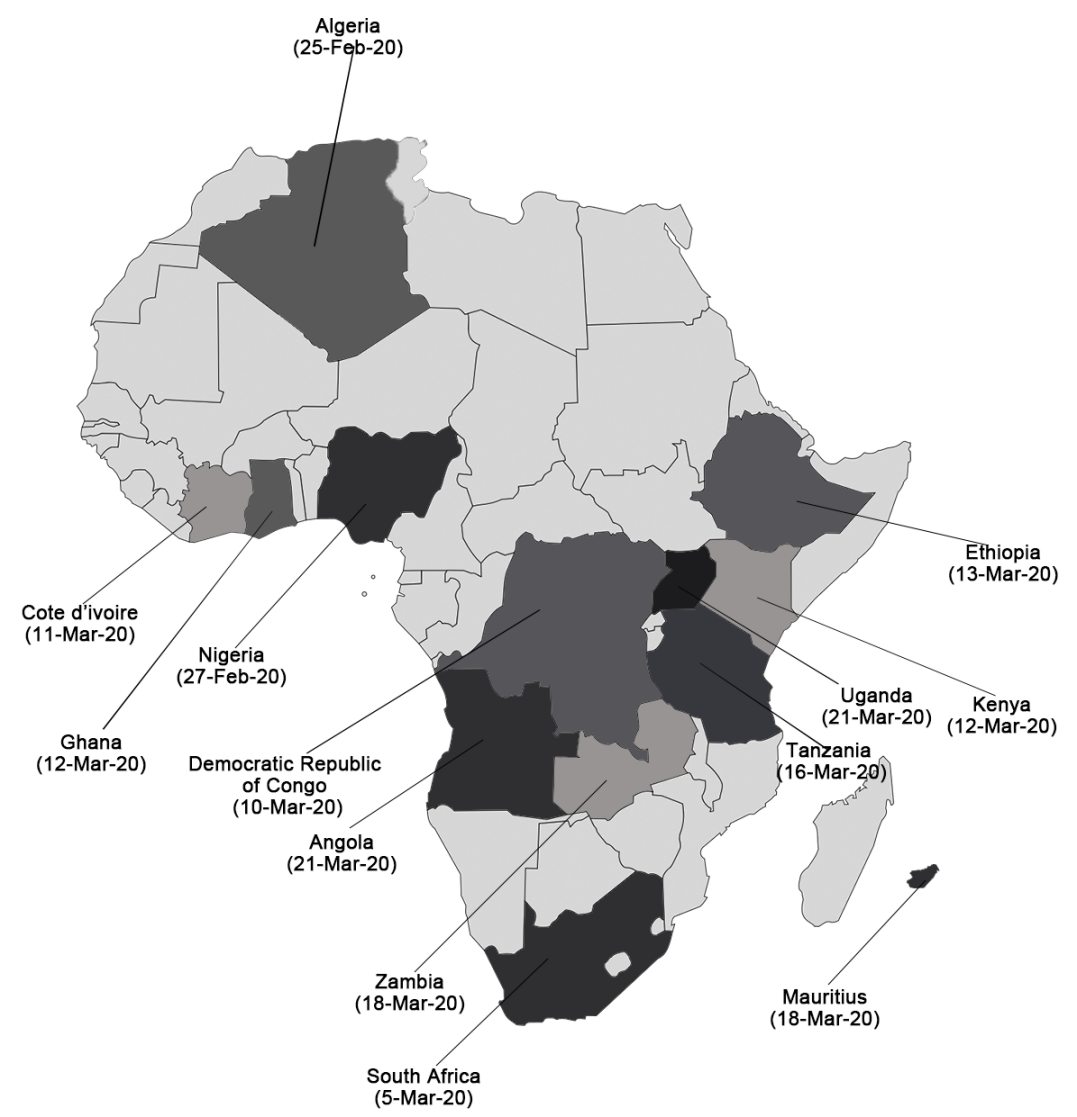
The 13 WHO identified priority countries for COVID-19 and date of index case(s)

We conducted a narrative review of data sources on RCCE to answer the aim of the study. Data reported in this review were obtained from reports, articles published in peer-reviewed journals, and grey literature available in the 13 priority African countries for COVID-19. The countries included Algeria, Ghana, South Africa, Tanzania, Kenya, Mauritius, Angola, Cote d’Ivoire, Ethiopia, the Democratic Republic of the Congo, Nigeria, Zambia, and Uganda (See Figure 1). Two researchers were involved in the independent review of literature to gather data for this study.

The inclusion criterion was basically data sources that provide information regarding COVID-19 RCCE responses and strategies in the predetermined African countries and were published between December 2019 and February 2021 while the exclusion criterion was any other data sources that do not provide information regarding COVID-19 RCCE responses and strategies in the predetermined African countries. We used both bibliographic and online search methods to collate the information and snowball further data. Our data included journal articles from different e-bibliographic databases, including MEDLINE, PubMed Central, PubMed, and Google Scholar.

The following main key search terms were used: “Community Engagement” “Outbreak Communication” “Risk Communication” “Social Mobilization” “Health Education” “Health Promotion” “Crisis communication” “COVID-19” 2019-nCoV, “SARS-CoV-2” “{+Each African Countries - Algeria, Ghana, South Africa, Tanzania, Kenya, Mauritius, Angola, Cote d’Ivoire, Ethiopia, the Democratic Republic of the Congo, Nigeria, Zambia, and Uganda}. The study team in consultation with experts in the field has surmised that much of the literature are not published in peer-reviewed journals because these are policy papers and not research studies, we expanded our search strategy to use search engines such as Google to locate and include these papers. Two members of the research team also conducted bibliographic searches to collate reports, newsletters, and government documents related to COVID-19 response activities in other to understand the RCCE strategies in the 13 African countries. The review of data sources was conducted in March 2021.

### COVID-19 risk communication and community engagement strategies in Africa

In order to support governments in developing RCCE strategies, the WHO issued an interim guidance on RCCE in March 2020.^10^ We used the framework from the interim guidance on the category of RCCE for COVID-19 to describe the strategies. The key categories of COVID-19 RCCE based on WHO interim guidance were risk communication systems, internal and partners coordination, community engagement, public communication, addressing infodemic, and training and capacity building.^10^ In Table 1, we summarized RCCE strategies as noted in the 13 African countries.

### Algeria

On 25 February 2020, the national health authorities in Algeria reported the first reported case of the outbreak.^14^ In response to the outbreak, WHO African region deployed a team of 7 people to Algeria for response capacity building, which includes RCCE training.^15^ The Senegal hub had in-depth discussions with the Algeria country team to support them in strategies to build capacity on RCCE.^16^ With the support of civil society partners and other stakeholders, the country continues to strengthen its response to the pandemic by enhancing RCCE.^17^

A set of risk communication materials (i.e., posters) have been developed and being disseminated through the support of government and volunteer organizations.^15^ Regular media updates were being provided by the Algerian Ministry of Health and Population team towards ensure effective RCCE.^15^ Through a dedicated COVID-19 website (http://covid19.cipalgerie.com/en/), social media channels (Facebook, Twitter, Instagram, and LinkedIn), key messages on back-to-school safety, important COVID-19 behaviours and COVID-19 vaccines were also made available.^16^ To address disinformation and misinformation, the Algerian government set up a COVID-19 free-phone (3030) as recommended by WHO, where people could speak to a trained health educator and gain more information.^15^ The country has also continued to engage in RCCE through working with youth-led organizations, communication platforms such as podcasts to reach more people, as well as awareness raising, education, and activism with various partners including regional and local stakeholders.^18^

Algeria is also making efforts to reach vulnerable members of the community using informational videos.^18^ The United Nations Population Fund (UNFPA) has also released a policy document on the need to engage the marginalized and vulnerable groups in COVID-19 RCCE in Eastern Mediterranean including countries like Algeria.^19^ For instance, the Algerian government is working with non-governmental organizations (NGOs) to disseminate COVID-19 advice and information to people living with HIV and people who use drugs through community engagements.^20^

### Angola 

On 21 March 2020, the Angola’s Ministry of Health announced the first two cases of COVID-19.^21^ In Angola, integrated infodemic management has been incorporated in its response to curb the outbreak.^22^ In July 2020, the COVID-19 Alliance was set up by the WHO office in Angola and the Ministry of Health, to fight infodemic.^22^ The Alliance also assisted in tracking and analysing conversations around the pandemic on social and traditional media and identify false and misleading information. Periodic updates and key messages about the disease were also published on its website (https://www.cisp.gov.ao:10443/en/), as well as using posters and infographics for information dessemination.^22^ The Ministry of Health in partnership with some agencies like UNICEF are also working together to strengthen RCCE in the country. For instance, according to UNICEF report, 1 082 506 people were reached with messages on COVID-19 prevention. Additionally, 12 855 people engaged through UNICEF social networks with messages on COVID-19 prevention. This report covered the period between 8 May and 22 May 2020.^21^

The Angolan COVID-19 helpline (111) was also leveraged to improve RCCE in the country,^21^ as well as community health mobilizers.^23^ Online training modules for social mobilizers on biosecurity measures, RCCE in times of COVID-19 were developed, tested, and implemented.^21,23,24^ The country is also making efforts to engage vulnerable communities in RCCE by working with partner organizations, community health mobilizers, NGOs and civil society organizations to disseminate COVID-19 information using tailor-made strategies like translation of information materials into local languages.^23,24^

### Cote d’Ivoire

On 11 March 2020, Cote d’Ivoire recorded its first case of COVID-19 with an Ivorian returning from Italy.^25^ The Government has implemented an emergency communication campaign to curb the spread of COVID-19.^26^ Communication lines (143, 144 or 101) have been established by the government, where the public can find out information about COVID-19.^26^

The country’s Ministry of Health and Public Hygiene website has been set up to provide extensive information to the public as well as training of health workers.^26^ COVID-19 campaigns are also being ramped up on social media (Instagram, Twitter, Facebook, and LinkedIn) and the government website (http://www.gouv.ci/Main.php), television and radio programs among others.^27^ Additionally, the Ministry of Health and Public Hygiene is continually updating the information on its web page about the measures that are being taken and urge the population to abide by official regulations to contain the spread of the virus.^28^

The national health authorities are also working with some organizations such as UNICEF to engage religious leaders, reporters, bloggers and media influencers, Voices of Youth, journalists, and U-Report communities among others.^27^ The country has also set up RCCE plans such as community outreaches and translation of COVID-19 information to local languages to engage vulnerable groups.^26^

### The Democratic Republic of the Congo

The first case of COVID-19 in the Democratic Republic of Congo (DRC) was a Congolese returnee from France, identified on 10 March 2020 in Kinshasa.^29^ Even though the country had experienced major Ebola outbreak in the past, scaling up RCCE to respond to COVID-19 was also paramount.^30^ The government, together with partners like UNICEF, set up COVID-19 hotlines.^31^ The hotline provides the opportunity for feedback alert mechanisms, correct information, and creates the link to medical assistance. The country also leveraged social media, young people, civil society organization leaders, and women leaders. These sectors could use the U-report platform through short messaging service (SMS) centers and automated bots for RCCE.^31^

For effective curbing of the pandemic, capacity building of the media professionals on prevention measures and warning signs of COVID-19 were also prioritized.^31^ Website (https://www.stopcoronavirusrdc.info/) and mass media such as radio and television were also used to reach more people with prevention messages on COVID-19.^31^ Door-to-door COVID-19 sensitization and engagement of religious leaders were also carried out to improve the reach of the message.^31,32^ The DRC’s Alerte Santé COVID-19 WhatsApp number was also set up for effective community engagement.^32^ However, there is paucity of data on how COVID-19 RCCE is implemented for the marginalized and vulnerable groups. The hero campaign intensified in Democratic Republic of the Congo to recognize and designate survivors as heroes and heroines and to reiterate that COVID-19 is real to address misinformation.^33^ Different organizations involved in curbing COVID-19 across DRC are involving multidisciplinary teams for COVID-19 sensitization.^34^

### Ethiopia

On 13 March 2020, the first confirmed COVID-19 case was reported in Ethiopia.^35^ In response to the outbreak, the Federal Ministry of Health started different containment activities such as RCCE to effectively curb the pandemic.^36^ One of the strategies used by the government of Ethiopia includes community mobilization and public sensitization as well as using toll free lines (8335, 952, etc.) to engage with the public.^37^ This was made possible with Ethiopia’s prevention-based primary public healthcare infrastructure and the health extension system that was established in last twenty years.^37^

About 79% of Ethiopia are inhabitant of rural areas with poor road network, weak transportation and communication links.^38^ RCCE task forces have been set up at the lowest administrative units and at health facilities to reach hard-to-reach communities.^36^ These units involve the country’s forty-two thousand health extension workers, with two per village, who undertake the task of sensitization and awareness creation.^36^ The religious leaders and young people were also engaged in the RCCE and updates are also being shared on government’s website (https://covid19.ephi.gov.et/) and social media.^40^ The country has also developed a RCCE plan that is inclusive of the vulnerable groups (people living with disabilities, children and women).^39^ The present prime minister has been making periodic briefings and public announcements COVID-19 pandemic.^40^ Ethiopian telecommunication corporation has also been using caller tune for COVID-19 precautionary measures awareness.^40^

### Ghana

The first two confirmed cases of COVID-19 outbreak in Ghana were reported on 12 March 2020.^41^ The Ministries of Health, Information and media instituted aggressive mass education and campaigns to create the necessary awareness in Ghana by working with partner organizations and national health authorities among others.^42^

Ghana’s RCCE strategies^43^ include series of meetings and executive briefings held with parliament and the media, relevant stakeholders, community leaders and thought leaders; and intensive sensitization on COVID-19 at the points of entry and catchment communities. Other strategies include development of communication support materials including bill-boards, printing of leaflets and pocket cards with quality control measures.Ghana is also leveraging on the development of broadcast and television documentaries and broadcast of informercials; social cultural, civic education and sensitization for religious organizations for systemic risk communication. Integration of COVID-19 epidemiological data into the weekly epidemiological bulletins and strengthening call/hotline centers across the country is also another Ghana’s RCCE strategy.

The governments also planned various approaches for community engagement including community-based surveillance, home visits and contact tracing including emergency line (121); risk communication for the general populations through a well-established networks of community health officers and community volunteers; sensitization and outreach services through community announcement centers and durbars; information sharing and counter misconceptions using government’s website (https://ghanahealthservice.org/covid19/) and social media; and sensitization and outreach services for the isolated, the quarantined and the vulnerable groups.^43^

### Mauritius 

The first three cases in Mauritius were announced on the 18 March 2020.^44^ Before those cases arrived, the Ministry of Health and Wellness started main sensitization programmes on COVID-19 on 23 January 2020 leveraging on media with partnership with key stakeholders for reaching impact.^44^ All pandemic-related information from the Government were from the National Communication Committee on COVID-19 via the daily press briefings in order to ensure effective information dissemination.^45^ Regular press conference provided the opportunity for timely, accurate and transparent information sharing, addressing misinformation, and urging stakeholders engagement in the response.^45^

Use of various feedback alert strategies, such as leveraging on the technicians from Ministry of Health and Wellness and the Police Force to relay daily feedback from the field to the high-level committee on COVID-19, to understand public perceptions and monitor the behaviour of the population.^6^ Different platforms for communication such as hotline (8924), daily press conferences, the Facebook pages and website, http://www.COVID19.mu, for maximum reach and to also meet the varied information-seeking behaviours of the different strata of the population.^46^ Mobile application *beSafeMoris* was also developed to frequently communicate to the public on issues pertaining to COVID-19.^44^ Fake news on social media was also sanctionable in order to address infodemic.^47^ Vulnerable persons especially the elderly and those suffering from chronic diseases such as diabetes and hypertension, were reached out to via both traditional and modern media to stay indoors.^46^

### Nigeria 

The Nigeria’s Ministry of Health announced the first COVID-19 case on 27 February 2020.^48^ The Presidential Task Force (PTF) on COVID-19 was set up by the President of the country on 9 March 2020, with a role to coordinate and oversee the Nigeria’s efforts to contain the outbreak and to curtail the untoward outcome of the pandemic in Nigeria.^49^ The PTF provided utmost strategic leadership to the country’s COVID-19 response guided by empirical evidence.^49^ Daily PTF media briefings were held to enlighten Nigerians on COVID-19 situation, emerging evidence, see to trending issues and provide updates regarding response of Nigerian government to COVID-19.^50^

Nigeria Centers for Diseases Control (NCDC) is leveraging on five strategies for its RCCE^51^ which include (1) dynamic listening and rumors management via media and social media surveillance, partners, stakeholders and using other social science tools; (2) communication engagement with affected communities directly or through influencers including awareness campaigns, setting up COVID-19 story blog (https://covid19blog.ncdc.gov.ng/), SMS, toll-free line (6232), community radio, interpersonal communication and using existing community engagement mechanisms; (3) public communication using modern and traditional media including websites (https://covid19.ncdc.gov.ng/); (4) internal and partner communication and coordination; and (5) a system to test the effectiveness of the RCCE. Efforts were also being made to engage the vulnerable groups in RCCE e.g., translation of COVID-19 information into local languages.^52^

### South Africa

The first COVID-19 cases in South Africa were detected on 5 March 2020.^53^ The government urged the populace to obey the precautionary measures put in place, including to stay indoors, avoid faith-based, social and sports gathering among others, to curb the impact of the outbreak in South Africa.^54^ Many COVID-19 awareness programmes were intensified to address pandemic-related discrimination and stigmatization, fear and disproving myths and misinformation about COVID-19 including the fifth generation wireless technology conspiracy theory.^55^

The Ministry of Health engaged media platforms such as television, radio and social media as well as SMS, toll-free lines (0800029999) leaflets, banners, government website (https://sacoronavirus.co.za/) and street campaigns for COVID-19 awareness.^54^ These platforms were engaged to teach the community about the effective ways of handwashing and the correct way to wear masks, sanitize their hands and observe physical distance among others.^54^ Partner organizations provided support to the National COVID-19 RCCE Technical Working Group to address the increased risk of transmission during festive periods through enhanced RCCE.^56^ The country is also making efforts to heighten RCCE for people living with HIV, people living with disabilities and other vulnerable groups by providing clear COVID-19-related information^57^

### Tanzania

Since the onset of the COVID-19 outbreak in Tanzania in March 2020,^58^ the WHO and its partners have been working closely with the national authorities to educate and actively communicate with the public about COVID-19 using multiple communications techniques.^59^

Later into the pandemic, COVID-19 response in Tanzania can be said to be worrisome.^60^ The COVID-19 denialism by Tanzanian government discourages RCCE.^60^ Tanzanian President John Magufuli was criticized globally for urging the public to continue to attend religious centres, rather than imposing heavy restrictions to curtail the spread of the virus.^61^The WHO has rebuked Tanzania for its ongoing lack of solidarity in the international fight against the COVID-19 outbreak when the country refuse to share COVID-19 statistics.^62^ Due to the government’s response to COVID-19, it is clear that the country lacks effective plans for RCCE, though partner organizations, civil society organizations and NGOs are making effort to implement and advance RCCE. Global health authorities are still hoping for Tanzania to strengthen its COVID-19 response.^61^

### Uganda 

Uganda recorded its first confirmed COVID-19 case on 21 March 2020.^63^ The first case motivated the Ugandan government to respond combatively by restricting public gatherings and imposing a movement restriction and a comprehensive travel ban into and out of Uganda, allowing only essential commodities.^63,64^ The president also held regular press briefings to manage infodemic and share situation reports about the outbreak.^65^ The country leveraged religious leaders for RCCE and shared update using government website (https://www.health.go.ug/covid/).^66^

The country employed various media platform such as radio, music, television, SMS messaging, Twitter, group emails, and WhatsApp messages to engage, mobilize, and sensitize the public on COVID-19 safety measures.^67^ A toll-free call (via 919) centre for COVID-19 response receives calls asking for information regarding the pandemic and alert for new cases.^65^ Standard operating procedures and guidelines were also shared periodically in electronic and print media on issues pertaining to the outbreak.^67^ WHO and the local health authorities were engaging community members and individuals in the high-risk villages and other vulnerable regions on COVID-19 emphasizing the consequences of not obeying precautionary measures.^68^

### Kenya

On 12 March 2020, the Kenya’s Ministry of Health reported the first case of COVID-19 in Nairobi.^69^ In response to the outbreak, the Federal Ministry of Health Kenya started different containment activities to effectively curb the pandemic.^70^ Kenya’s RCCE strategies^71^ include (1) strengthening information and coordination management structures across Kenyan ministries as well as the county governments; (2) promoting key public health campaign information and ensuring conversations with the people to curb the spread and transmission of the virus among general and vulnerable groups; (3) engaging formal and informal leaders to share correct information; (4) promoting 2-way communication with communities to reduce pandemic-related fear, infodemic, and discrimination; (5) ensuring tailor-made COVID-19 information to various audiences and channels; (6) ensuring health workers have the capacity to effectively provide accurate COVID-19 information; and effective monitoring and evaluation strategies for measuring outcomes at all levels. Different communication channels such as mainstream media, social media, community dialogue, government website (https://www.health.go.ke/), toll-free line (719) and education-entertainment among other have been leveraged for RCCE in Kenya.^71^

### Zambia

Zambia announced an outbreak on 18 March 2020, after the first two cases of COVID-19.^72^ The response of the Zambia government has been proactive, with a focus on effort to ensure precautionary measures are observed at the individual and population strata.^73^ The government is also making efforts to keep the public abreast of the status of the pandemic through periodic press briefings. During the briefings, the press corps were provided the opportunity to ask questions which were being addressed publicly.^72^ Zambia was also leveraging radio, toll-free line (909), television programme, social media, website (https://www.moh.gov.zm/), posters, pocket cards, fliers and bill-board campaign in coordination with partner organizations.^73^Sector partners mapped densely populated areas, areas with populations more than hundred people, region with major vulnerable groups, markets, burial yards, door-to-door campaign using evidence-based planning tool called Geo-Referenced Infrastructure and Demographic Data for Development 3 Mapping. This resulted in identification of areas that needs more attention in terms of response activities and response team will be deployed for sensitization and awareness raising.^73^

### Challenges facing COVID-19 RCCE in the 13 African countries

We identified unique challenges facing COVID-19 RCCE in the selected African countries which are also applicable to other African countries.

### Distrust in government

Long-standing political corruption motivates widespread distrust in governments and this discourages people’s cooperation and heed to government protocols which has major untoward impact on responses to COVID-19 and facilitates the spread of the coronavirus.^74^ This fundamental lack of trust in government has untoward impacts on RCCE and this is not an uncommon challenge in Africa to public health responses.^75^ For instance, a study has shown how political distrust has continue to impact RCCE in Nigeria.^74^ Another study in Democratic Republic of Congo revealed on how previous corruption practices by the government in response to Ebola have impacted public trust resulting into negative impacts on COVID-19 RCCE.^76^ Similar cases of how distrust in government has impacted COVID-19 RCCE in Zambia,^77^ Cote d’Ivoire,^78^ Ghana,^79^ Kenya,^80,81^ Uganda,^81^ and across other African countries^82^ have been reported.

### Weak healthcare systems

Due to limited resources and weak health systems,^83^ the COVID-19 pandemic presented African countries with new challenges: the need to strengthen RCCE mechanisms. Effective RCCE in some African countries is hampered by limited resources, limited skilled staff, and poor coordination as well as limited funding and lack of plans and guidelines.^13^ Even though WHO, Africa CDC and other stakeholders are investing in RCCE training,^1^ the efforts and resources invested should have been channeled to other areas of responses, if the government had invested in public health emergencies RCCE prior to the pandemic.

Lack of clear roles of partners in RCCE is also another challenge that can be attributed to feeble health systems on the continent.^84^ While RCCE plays a key role in outbreak response, poorly defined stakeholder roles can prove to be ineffective.^85^ African countries also have low healthcare workforce^86^ and limited experience in coordination and reporting^87^ which are crucial to effective RCCE. Africa is also facing double burden of communicable and non-infectious diseases and the need to ensure RCCE for these diseases, together with COVID-19, is challenging. High levels of poverty, uneven access to healthcare services across regions in the same country making some areas more vulnerable than the others and lack of RCCE response infrastructure can also make containment efforts challenging.^83^ All these can be attributed to longstanding lack of investment in health systems resulting into weak healthcare systems on the continent.

### Widespread rumors and misinformation

In a cross-sectional survey among 1969 respondents conducted in different African countries (Kenya, Cameroon, Ghana, Tanzania, Nigeria, South Africa, and Uganda), the study revealed that about 19% believed that the pandemic was designed to reduce world’s population, 22% thought the ability to seize your breath for ten seconds meant that you do not have COVID-19, 28% believed coronavirus can be washed down by drinking hot water, and 14% thought that COVID-19 had minimal effect on Blacks in contrast to Whites.”^88^ The infodemic continues to undermine COVID-19 RCCE in many African countries which is further worsened by social media.^88^ Some people still doubt the existence and nature of the outbreak and ignore safety precautions.^89^ Some of the myths, rumors and misinformation reported in Zambia, Nigeria and some African countries include “The COVID reports are fabricated”, “It’s a strategy to get rid of Africans”, “Young people are immune to the pandemic”, “Communities without soap say maybe they can use very hot water for washing hands”, “People say masks are not meant for them but for a particular class of people and race”, “COVID-19 is cured by drinking ginger tea”, “It is a disease of the politicians”, “The people involved are just doing it for money’s sake”, “COVID is for the rich”, and “God is annoyed with humans because they have abandoned Him by stopping to gather for worship” among others.^89^

On the other hand, the case of Tanzania is different in that the government is the one promoting COVID-19 denialism and spreading unscientific claims about the pandemic which is hampering effective RCCE in the county.^60^ National health authorities need to move towards 2-way feedback mechanism, strengthen social media surveillance to debunk rumours and misinformation and to inculcate effective community engagement practices in local formats, languages and channels to effectively curb infodemic.^30^

### Exclusion of some vulnerable groups

Despite the efforts made by African governments to ensure an inclusive response to COVID-19, the uniform response efforts continue to exclude the specific needs of vulnerable groups e.g. people living with disabilities.^90^ A study revealed that only 54% of sub-Saharan African countries, including Ethiopia, Nigeria, Ghana and Zambia among others, have a sign language interpreter available in COVID-19 press conferences and briefings.^91^ This implies that one of the goals of RCCE to provide clear information to all is hampered. COVID-19 has revealed the need to invest and ensure all-inclusive RCCE in addressing public health emergencies on the continent.^90^ It is important to support RCCE coordination and activities to include local stakeholders, particularly faith-based leaders, religious organisations, grassroot organisations, and groups representing at-risk populations such as people living with chronic diseases and disabilities.^30^

### Resistance and Inertia

A key challenge to RCCE in African countries includes resistance and inertia, with the etiology ranging from cultural to religious. In response to COVID, the public have been asked to adopt a change in their lifestyles and usual practices, or sacrifice time and other resources to reduce transmission. These changes have faced a backlash.^92^

For instance, Kenya,^93^ South Africa,^94^ and many other African countries have a communal culture, and this is responsible for resistance to presence guidelines on burials and other measures. Similar resistance has been seen in obeying precautionary measures to stop handshake and body hug in some African countries.^94^ Congregations of the faithful is practiced by many religions which increase transmission risk of the virus.^95^ The social, cultural, and religious resistance have posed challenges to effective RCCE in many African countries. Collaborative intervention to work on feedback from the community will be advantageous in terms of public acceptance of COVID-19 response measures because such efforts will enhance trust and inform effective RCCE.^30^ Engaging cultural/religious leaders and health experts on the same ground to formulate the guidelines to social engagements may help reduce the resistance to change.

## Conclusion and recommendations

Most of the WHO-prioritized African countries have RCCE strategies to curb the spread of COVID-19. Given the common RCCE approaches and interventions seen across the continent, it is clear that countries are learning from each other and from global health organizations to develop RCCE programs for COVID-19. However, the RCCE response activities were not without challenges, which included distrust in government, cultural, social, and religious resistance, and inertia, as well as widespread of fake news and rumors, exclusion of vulnerable populations and longstanding issues of weak healthcare systems.

We recommend strengthening strategic mapping of partners, investing in proper coordination structures, resources, and training, improving public trust through effective leaderships, ensuring adequate planning, and strengthening documentation and reporting of activities and experiences for RCCE in Africa. It is also important to assess existing RCCE structures and to ensure approaches to support contextually distinct, acceptable, and appropriate structure for future diseases outbreaks. The African countries need to integrate data and models in their approach to RCCE to strengthen these efforts. Further investments are needed in enhancing knowledge sharing and resilience across countries and digitalized systems for monitoring isolation/quarantine and tracking for RCCE.

## Funding

YAA is a recipient of 2020 Royal Society of Tropical Medicine and Hygiene and National Institute of Health Research UK Small Grant Award. The authors undertake this work with the funding support from Royal Society of Tropical Medicine and Hygiene and National Institute of Health Research UK Small Grant Award.

## Competing interests

None.

## Ethical approval

Not applicable.

## Authors’ contributions

YAA led, conceptualized, and wrote the paper. YAA performed the literature review with the support from AR. DELP and AR supervised and critically reviewed the manuscript. All authors have read and agreed to the final version of the paper.

**Table 1:** Summaries of RCCE strategies in the 13 African countries

**WHO category of COVID-19 RCCE strategies**	**Identified strategies by the African countries**
Risk communication systems	-Engagement of highest level of government in the risk communication system from the national level to the local community level -Strengthening the existing risk communication systems prior the pandemic or setting up the risk communication plans to respond to the pandemic -Setting up RCCE operational team and working groups -Budget planning for RCCE
Internal and partners coordination	-Identifying and working with partner organizations and agencies such as UNICEF, to enhance RCCE -Setting up standard operating procedures and RCCE coordination by the national health authorities.
Community engagement	-Leveraging community influencers, community health workers and religious leaders -Engagement through modern and traditional media including radio, TV, SMS, social media, etc. -Using hotlines as a tool for community engagement -Identifying target audiences for community engagement and addressing communication needs for the vulnerables e.g., elderly and people living with disabilities -Initiatives to understand the concerns, attitudes, and beliefs of diverse and specific audiences.
Public communication	- Leveraging spokesperson, community influencers, health professionals, community health workers and religious leaders - Leveraging modern and traditional media platform including radio, TV, SMS, social media, etc.
Addressing infodemic	Leveraging information technology, social media, healthcare professionals, community leaders, religious leaders to address rumors and misinformation.
Training and capacity building	Trainings on RCCE for relevant stakeholders e.g., healthcare professionals, operational teams, etc.

We also describe in details the RCCE approaches in the 13 WHO-prioritized African countries below.
